# Construction of Genetic Linkage Maps From a Hybrid Family of Large Yellow Croaker (*Larimichthys crocea*)

**DOI:** 10.3389/fgene.2021.792666

**Published:** 2022-01-03

**Authors:** Xinxiu Yu, Rajesh Joshi, Hans Magnus Gjøen, Zhenming Lv, Matthew Kent

**Affiliations:** ^1^ Department of Animal and Aquacultural Sciences, Faculty of Biosciences, Norwegian University of Life Sciences, As, Norway; ^2^ National Engineering Research Centre of Marine Facilities Aquaculture, Marine Science and Technology College, Zhejiang Ocean University, Zhoushan, China; ^3^ GenoMar Genetics AS, Oslo, Norway

**Keywords:** large yellow croaker, RAD sequencing, linkage map, collinearity, recombination rate

## Abstract

Consensus and sex-specific genetic linkage maps for large yellow croaker (*Larimichthys crocea*) were constructed using samples from an F_1_ family produced by crossing a Daiqu female and a Mindong male. A total of 20,147 single nucleotide polymorphisms (SNPs) by restriction site associated DNA sequencing were assigned to 24 linkage groups (LGs). The total length of the consensus map was 1757.4 centimorgan (cM) with an average marker interval of 0.09 cM. The total length of female and male linkage map was 1533.1 cM and 1279.2 cM, respectively. The average female-to-male map length ratio was 1.2 ± 0.23. Collapsed markers in the genetic maps were re-ordered according to their relative positions in the *ASM435267v1* genome assembly to produce integrated genetic linkage maps with 9885 SNPs distributed across the 24 LGs. The recombination pattern of most LGs showed sigmoidal patterns of recombination, with higher recombination in the middle and suppressed recombination at both ends, which corresponds with the presence of sub-telocentric and acrocentric chromosomes in the species. The average recombination rate in the integrated female and male maps was respectively 3.55 cM/Mb and 3.05 cM/Mb. In most LGs, higher recombination rates were found in the integrated female map, compared to the male map, except in LG12, LG16, LG21, LG22, and LG24. Recombination rate profiles within each LG differed between the male and the female, with distinct regions indicating potential recombination hotspots. Separate quantitative trait loci (QTL) and association analyses for growth related traits in 6 months fish were performed, however, no significant QTL was detected. The study indicates that there may be genetic differences between the two strains, which may have implications for the application of DNA-information in the further breeding schemes.

## Introduction

Large yellow croaker (*Larimichthys crocea*) has become an important aquaculture species in southeast China, where Mindong and Daiqu are the two major strains farmed. Artificial breeding of the Mindong strain started in 1985, while the Daiqu strain has been bred since 1999 (Chen et al., 2018). In 2019, the total production of large yellow croaker exceeded 220,000 tons and accounted for more than 12% of the cultured marine fish production of China (Yu X. J. et al., 2020).

The production is supported by several breeding programs, of which the majority are based on classical basic selection methods, like phenotypic selection (Chen et al., 2018). However, modern breeding approaches, such as marker assisted selection (MAS), can further enhance the genetic gain for economically important traits. MAS can greatly increase the efficiency if a sufficiently large QTL is detected, typically through QTL linkage mapping and association studies (Zenger et al., 2019). An outstanding example of MAS applied in aquaculture was the discovery of a QTL imparting resistance to infectious pancreatic necrosis (IPN) in Atlantic salmon, accounting for about 80% of the total variation in this trait (Moen et al., 2009; Houston et al., 2012). Information from the QTL was used in selective breeding to generate IPN resistant fish, which now dominate production in Norway, leading to a remarkable reduction in IPN outbreaks (Norris, 2017). Subsequent studies have provided functional genomics data indicating that mutations in the epithelial cadherin gene (cdh1) affect virion internalization (Moen et al., 2015), demonstrating the power of genomic tools to help reveal the mechanistic basis for important traits. Although MAS can be useful for some traits where major QTLs have been identified, most traits of economic importance in aquaculture species (i.e., production traits) are assumed to be polygenic, and often have low-to-moderate heritabilities (Zenger et al., 2019). As a result, application of MAS to improve these complex traits may be inefficient. For such polygenic traits, *genomic selection* is a viable alternative, based on genomic breeding values predicted on a genome-wide scale, allowing even small QTLs to contribute (Meuwissen et al., 2001). For large yellow croaker, the estimates of heritability for body weight (0.31 ± 0.06), body length (0.33 ± 0.06) and body height (0.41 ± 0.07) in 6 months fish, and the genetic correlations between them ranged from 0.74 to 0.95 (Yu X. X. et al., 2020).

High-throughput sequencing has transformed genetics by making it relatively easy to generate genome-wide genetic marker datasets, which are a prerequisite for QTL identification in MAS. Significant progress was made through the discovery of cost-effective restriction-site associated DNA sequencing (RADseq) based strategies (Baird et al., 2008). RADseq can generate medium density SNP resources and has been successfully used in various fish species for genetic linkage maps, QTL analysis and population genetics (Davey and Blaxter, 2010), e.g., in Atlantic salmon (Houston et al., 2012; Gonen et al., 2014), channel catfish (Li et al., 2014) and Nile tilapia (Palaiokostas et al., 2013).

Several genetic linkage maps for large yellow croaker have been developed using different approaches (Supplementary Table S1). The first two genetic linkage maps made publicly available were constructed using amplified fragment length polymorphism (AFLP; Ning et al., 2007) and simple sequence repeats (SSR; Ye et al., 2014). However, next-generation sequencing technologies have made detection of large numbers of genome-wide SNP markers relatively easy, and Ao et al. (2015) constructed a SNP genetic linkage map with a total length of 5451.3 cM using RADseq, while Xiao et al. (2015) constructed a genetic map of 2,632 cM using RNA sequencing (RNAseq) of expressed genes. More recently, Kong et al. (2019) constructed a double-digest restriction-site associated DNA (ddRAD) based genetic map using 5261 SNPs with a total length of 1885.67 cM. Despite using different approaches, these SNP linkage maps have one thing in common, as they were all developed using only the Mindong strain.

Daiqu strain of large yellow croaker has been successfully cultured since 1999 and the aquaculture production is on an industrial scale (Chen et al., 2018). Most consumers prefer lean large yellow croaker, and the body shape has become an important economic trait (Dong et al., 2019). The Daiqu strain has better performance for this trait, as the ratio of body length and body height is significantly higher than for the Mindong strain (Huang et al., 2006). The Daiqu strain also has later sexual maturation and better tolerance to lower temperatures than the Mindong strain (Liu and Mitcheson, 2008; Miao et al., 2014). The offspring from a crossing between Mindong and Daiqu displayed significant heterosis in body shape and growth of fish after 526 days (Li et al., 2010). Our study therefore sought to develop a genetic linkage map in a crossed (F_1_) family arising from these strains.

The aim of this study was to construct consensus and sex-specific linkage maps based on a hybrid family from the Daiqu and Mindong strains using RADseq, to compare the linkage map to the latest physical map (*ASM435267v1*), and to perform a QTL analysis and association analysis for growth-related traits.

## Materials and Methods

### Mapping Family

A female (F_3_ of wild Daiqu strain, from an aquaculture farm in Xiangshan, Zhejiang province) and a male (approx. F_10_ of wild Mindong strain, from an aquaculture farm in Fuding, Fujian province) large yellow croaker were crossed to generate a fullsib family (Yu X. et al., 2017). One-hundred and twenty offspring were randomly selected at 6 months, and the following growth traits were recorded: body weight (BW), body length (BL) and body height (BH). Fin clips were preserved in 99% ethanol and sent to BGI Genomics Company (Shenzhen, China) for sequencing.

### RAD Sequencing and SNP Calling

Library preparation was performed by BGI according to Baird et al. (2008). In brief, individual genomic DNA samples were digested using the restriction enzyme *Pst I*, and the resulting fragments were ligated to a double-stranded Illumina sequencing primer containing a sample-specific barcode sequence. Libraries were then pooled and sheared by sonication, and fragments from 300–500 bp were separated by agarose gel electrophoresis and purified before ligating a Y-adapter to the sheared ends. Fragments including both barcode and Y-adapters were amplified with PCR to generate the final RAD libraries, which were then sequenced using a *Hiseq2000* platform to produce paired-end reads.

Raw reads were processed by BGI using the *Reseqtools* software package (https://github.com/BGI-shenzhen/Reseqtools) to remove adapter sequences and low-quality reads, and to de-multiplex the pool. The retained reads were analysed and genotyped using *Stacks* (Catchen et al., 2013) and in-house analysis pipelines, and RAD-tags with too low (<2) or too high (>100) sequencing coverage were excluded.

### SNP Filtering and Linkage Map Construction

SNPs missing in >10% of samples and minor allele frequency (MAF) < 0.05 were excluded using the PLINK software (Purcell et al., 2007). Markers were individually tested against the expected segregation ratio, based on parental genotypes, and those showing significant segregation distortion (*p* < 0.05, *χ* ^2^ test) were removed by PLINK.

The remaining SNPs were used to generate consensus and sex-specific maps using Lep-MAP2 software (Rastas et al., 2013). All SNP markers that passed filtering (n = 20,186) were used to produce the consensus map, while those markers polymorphic in the father (n = 11,684) or mother (n = 11,838) were used to construct their respective sex-specific linkage maps. LGs were developed using the *separate chromosomes* module, with a logarithm of odds (LOD) score ranging from 1 to 20. A LOD score of 9, which gave 24 LGs and the lowest number of single markers, was finally selected. The option *sizeLimit =100* was used to generate linkage groups of size ≥100 markers. The module *JoinSingles* could not assign any of the singular markers to any of the 24 LGs. Eventually, 20,147 SNPs were ordered using the *OrderMarkers* module, which assign the markers with paternal or maternal positions for the sex-specific maps. The option *sexAverage = 1* was applied during execution of *OrderMarkers* to get positions for the consensus map. To avoid the map distances being too long, especially when the number of markers per chromosome was much higher than the number of individuals, the parameter *minError = 0.15* was used. Finally, the *Kosambi* mapping function was used to calculate genetic distance between markers. The LG were numbered by the SNP size of each LG (i.e., the LG with the largest SNP number was labelled LG1). Illustrations of the consensus and sex-specific linkage maps were drawn using MapChart 2.32 (Voorrips, 2002).

### QTL Analysis and Association Analysis

QTL analysis was initially performed using the QTL *IciMapping* software by the option *inclusive composite interval mapping with an additive effect* (ICIM-ADD) (Li et al., 2015; Meng et al., 2015). The LOD threshold for QTL significance of each trait was determined by a permutation test (1,000 replications) with a genome-wide significance level of 0.05. The permutation threshold method for QTL mapping estimates the null distribution of the genome-wide maximum LOD score by shuffling the phenotypes relative to the genotype data, breaking the association between the phenotype and the genotypes (Churchill and Doerge, 1994). The genome-wide LOD thresholds are calculated based on the 1-α quantiles of the genome-wide maximum LOD scores obtained from the permutations, where *α* is the significance level (*α* = 0.05 in our case).

As a complementary method for QTL mapping, a genome-wide association study (GWAS) was performed using SNPs subjected to a more stringent quality filtering than that was applied for linkage mapping to ensure a high QTL identification accuracy. Using PLINK, individuals displaying more than 5% missing genotypes were removed. Also, SNPs were removed in cases where missing genotypes >5% across samples and Hardy-Weinberg *p* value (Fishers exact test) < 10^–9^. The final SNP set used for GWAS thus included 16,570 SNPs from 74 individuals.

The genome-wide association analysis was performed using a mixed linear model equation on BW, BL and BH by the *Genome-wide Complex Trait Analysis* (GCTA) program, with the *-mlma* function (Yang et al., 2011). The following model was used:

y=a+bx+g+e

where y is the phenotypes (BW, BL, BH), a is the overall mean for each trait, b is the additive genetic effect of the candidate SNP to be tested for association, x is the incidence matrix for the candidate SNPs, g is the polygenic effect and e is the vector of random residual effects.

SNPs were considered genome wide significant when exceeding the Bonferroni threshold for multiple testing (*α* = 0.05) of 0.05/tg = 3.017502 × 10^–6^, where tg = 16,570 (total number of genome-wide SNPs); and SNPs were graded as chromosome-wide significant when Bonferroni threshold for multiple testing (*α* = 0.05) surpassed 0.05/tc = 7.246377 × 10^–5^, where tc = 690 (average number of SNPs per chromosome). The genome-wide significant threshold used in this study was *p* ≤ 3.017502 × 10^–6^ (−log10 (P) = 5.52), while chromosome-wide significant threshold was *p* ≤ 7.246377 × 10^–5^ (−log10 (P) = 4.14). SNPs were visualised along the linkage groups using the Manhattan function in the R package QQMAN (Turner, 2014).

### Collinearity Analysis: Genetic vs. Physical Map

To explore the level of agreement between our consensus genetic map and a recently published physical map, the large yellow croaker assembly, *ASM435267v1* (GenBank ID GCA_004352675.1), the RAD-tag sequences (82 bp) from the consensus linkage map were aligned to *ASM435267v1* using BLASTN (https://blast.ncbi.nlm.nih.gov/Blast.cgi) with the following parameters: *expect value e ≤ 1 × 10* ^*–15*^ *, identity ≥ 95%, matched length ≥ 81 bp, mismatches ≤ 1* and *gap open = 0*. If a query sequence hit two or more loci in the physical assembly and the difference between the 1st and 2nd smallest e-values was greater than 10^3^, the 1st smallest e-value was chosen to define the hit. Finally, 9885 SNPs from the consensus map hit the physical map.

The relative positioning of RAD sequences in the genetic map and the physical map were graphically presented using *shinyCircos* (Yu Y. et al., 2017). The marker positions on genetic map were multiplied by 4 × 10^5^ for better visualisation of the Circos plot.

### Adjusting Genetic Maps Based on the Physical Map

The collinearity analysis highlighted 107 SNPs whose assignment to LGs disagreed with their physical assignment to chromosomes. Examples of this were seen in all LGs and, when detected, SNPs were reordered according to the physical map. The adjusted SNP order in each linkage group was used as an input of *evaluateOrder* option in the *OrderMarkers* module of Lep-MAP2, and the genetic distances were recalculated using the *Kosambi* mapping function. The integrated consensus and sex-specific linkage maps were drawn using *MapChart 2.32* (Voorrips, 2002).

Scatter plots were generated between the integrated linkage maps in cM distances and the physical map in Mb distances by using the *ggplot2* package in R. Recombination rates throughout the genome in the integrated female and male genetic maps were estimated using *MareyMap* online (Siberchicot et al., 2017) with a computed sliding window size of 3.37 Mb. The threshold markers number in a window was set to 8, the default value. The recombination rate changes throughout the genome in the integrated female and male maps were visualised by using the *ggplot2* package in R.

## Results

### Sequencing and SNP Filtering

Approximately 1.9 billion reads were produced after sequencing two parents and 120 offspring, with each individual contributing roughly 15 ± 2.9 million reads. After reads filtering and RAD-tag SNP detection, approximately 370,000 variants were detected within each individual. The average heterozygosity rate was 32.5%. After filtering for segregation errors, MAF and missing genotypes, a final set of 20,186 SNP markers was used for linkage map construction.

### Linkage Map Construction

**FIGURE 1:**
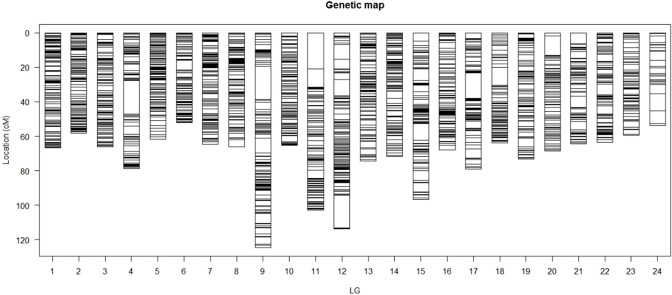
**|** The consensus linkage map for large yellow croaker. The dark bands show the density of the SNPs in the region of the LGs, whereas white bands show the regions with no SNPs.

The SNPs were assigned to 24 LGs, in accordance with the haploid chromosome number (Lou et al., 2015). The consensus map (Figure 1; Table1) covered 1757.4 cM, with individual linkage group lengths ranging from 51.9 cM (LG6) to 124.6 cM (LG9). The number of markers per linkage group varied from 243 to 1,230, with an average genetic distance between markers of 0.09 cM and a standard deviation of 0.037 (Table1).

**TABLE 1:** **|** Key figures for the genetic linkage maps of large yellow croaker.

LG	Consensus			Female		Male	
	No. of markers	Size (cM)	Average distance (cM)	No. of markers	Size (cM)	No. of markers	Size (cM)
1	1,230	66.8	0.05	613	78.2	745	51.8
2	1,125	58.3	0.05	783	65.7	716	43.1
3	1,029	66.0	0.06	660	86.7	574	55.5
4	1,000	78.7	0.08	654	66.8	503	83.5
5	997	61.8	0.06	626	72.6	629	49.4
6	973	51.9	0.05	543	54.2	548	43.8
7	967	64.6	0.07	532	67.2	558	50.1
8	949	66.3	0.07	458	70.2	647	60.4
9	899	124.6	0.14	609	68.3	375	58.0
10	875	65.2	0.07	484	68.6	505	49.4
11	850	102.8	0.12	475	64.4	468	51.8
12	837	113.7	0.14	533	68.6	410	59.3
13	831	74.4	0.09	480	58.5	480	52.8
14	817	71.9	0.09	589	71.8	377	50.7
15	814	96.6	0.12	297	70.4	589	52.9
16	791	68.0	0.09	387	61.2	526	55.2
17	789	79.0	0.10	417	70.4	476	45.6
18	789	63.9	0.08	616	55.2	566	48.6
19	787	73.2	0.09	589	54.2	283	56.6
20	741	68.4	0.09	539	54.2	358	46.1
21	693	64.5	0.09	378	50.5	392	55.3
22	689	63.6	0.09	237	39.8	528	58.0
23	432	59.3	0.14	225	65.7	273	50.5
24	243	53.9	0.22	114	49.8	158	50.8
Total	20147	1757.4	0.09	11838	1533.1	11684	1,279.2

Separate male and female maps were constructed using segregating (heterozygous) markers from each parent. The total length of the male linkage map was 1,279 cM, and the total length of the female map was 1,533 cM (Table1, Supplementary Figure S1). In the female map, LG length ranged from 39.8 cM (LG22) to 86.7 cM (LG3), and the SNP number per LG varied from 114 to 783. In the male map, the length of each LG varied from 43.1 cM (LG2) to 83.5 cM (LG4), and the SNP number in each LG varied from 158 to 745. The average distance between markers for female and male is thus 0.11 and 0.13 cM, respectively. The female-to-male length ratio ranged from 0.7 (LG22) to 1.6 (LG3), with an average of 1.2 ± 0.23; most LGs in the female map were larger than those in the male map, with the exceptions of LG4, LG19, LG21 and LG24.

### QTL Analysis and Association Analysis for Growth Traits

The growth traits, BW, BL and BH, recorded in 120 offspring at 6 months of age, are presented in Table 2. In the QTL analysis, the LOD threshold used was 8.81 for BW, 7.35 for BL, and 18.71 for BH. However, no QTL was above the LOD threshold for any of the growth traits, BW, BL or BH (Supplementary Figure S3). In the GWAS analysis, the estimated genomic heritabilities for the three traits were close to zero (Table 3). A total of 16,570 SNPs from 74 recorded individuals were used, however, no SNPs crossed the genome or chromosome-wide significant level (Supplementary Figure S4).

**TABLE 2:** Mean ± SD, range and coefficient of variation (CV) of growth traits at 6 months.

	Body weight, g	Body length, cm	Body height, cm
Mean ± SD	45.2 ± 13.8	13.8 ± 1.6	3.6 ± 0.5
Range	20.8–89.3	9.5–17.7	2.8–7.6
CV (%)	31	11	15

**TABLE 3:** Estimates of variance components and heritability with standard errors (in parenthesis) using the genomic relationship matrix in GWAS by GCTA.

Traits	$\sigma_{g}^{2}$	$\sigma_{e}^{2}$	$\sigma_{P}^{2}$	$Genomic\;h^{2}$
BW	0.00022 (48.66)	194.5570 (42.94)	194.5572 (35.96)	0.000001 (0.25)
BL	0.000003 (0.69)	2.925069 (0.70)	2.925072 (0.52)	0.000001 (0.24)
BH	0 (0.08)	0.377716 (0.09)	0.37716 (0.07)	0.000001 (0.22)

$\sigma_{g}^{2}$ , Genetic variance; $\sigma_{P}^{2}$ , Phenotypic variance; $\sigma_{e}^{2}$ , Residual variance; $h^{2}$ , Heritability.

### Collinearity Analysis

**FIGURE 2:**
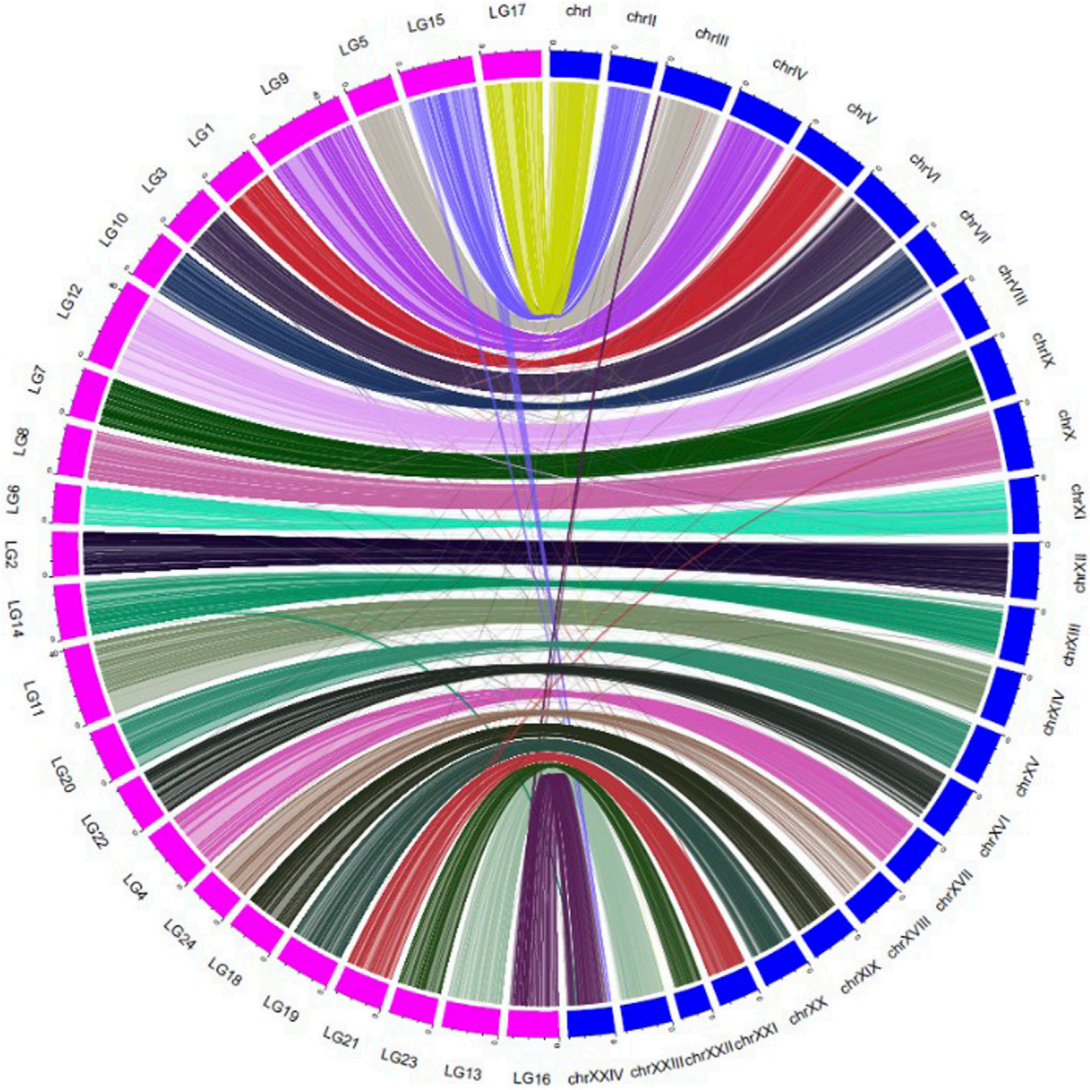
Collinearity between the consensus genetic map (LG1-24) and the physical map (chr I-XXIV) of large yellow croaker.

In total, 9885 SNPs from the consensus map hit the physical map (*ASM435267v1*), but there were 107 SNPs hitting non-corresponding chromosomes. A collinearity analysis, comparing the consensus linkage and physical maps, was performed (Figure 2). The average correlation coefficient between the genetic map and the physical map was 0.78 ± 0.16 (Supplementary Table S2). Each LG matches well with its corresponding chromosome of the physical map, with an average matching percentage of 98.92 ± 1.5%. There were 7 LGs that showed no mismatch between the genetic map and the physical map; LG7, LG9, LG10, LG17, LG18, LG20 and LG24 (Supplementary Table S3).

### Integration of Physical and Genetic Maps

**FIGURE 3:**
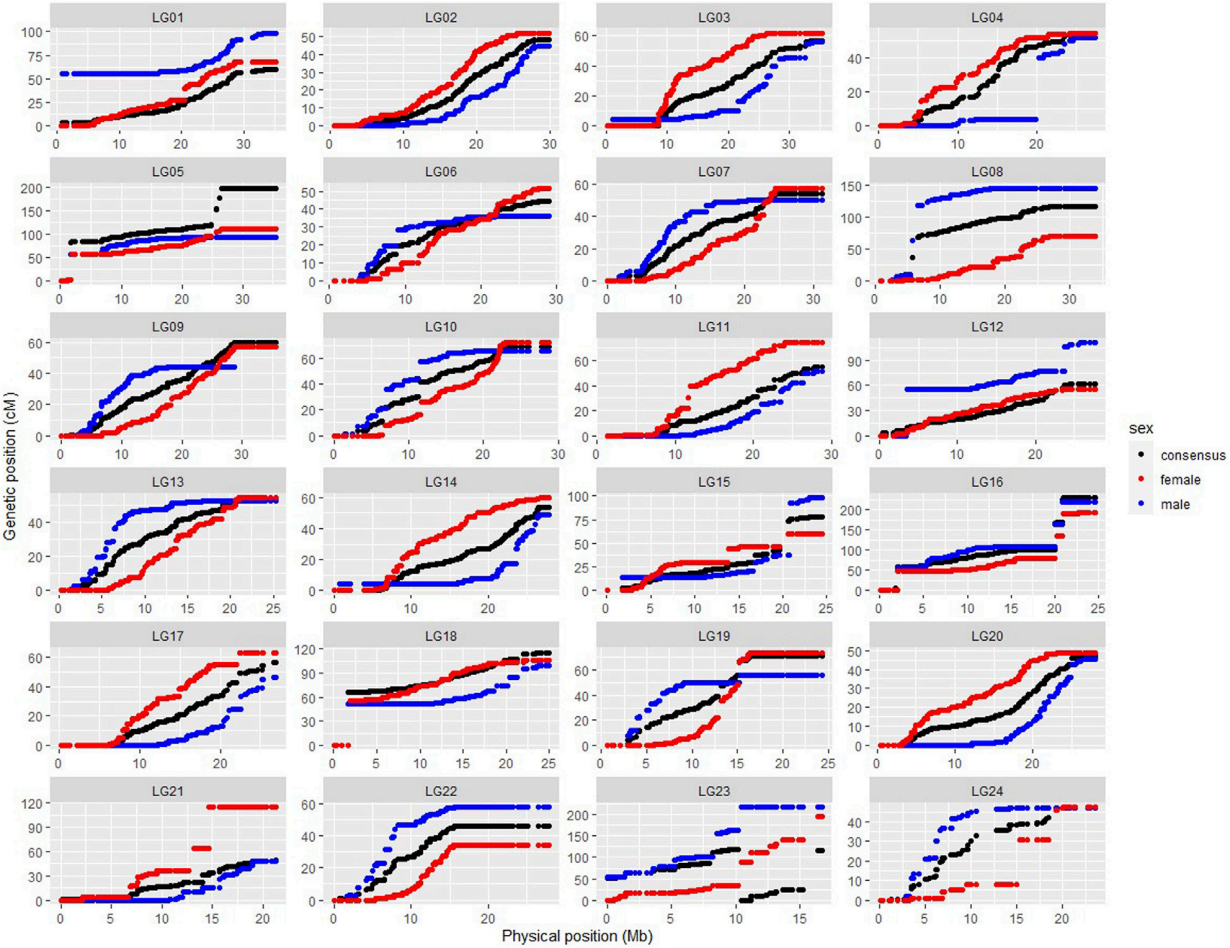
Scatter plots showing SNP linkage map positions (cM; *y*-axis) versus physical positions (Mb; *x*-axis) for integrated female (red), male (blue) and consensus (black) genetic maps. (Fragmented linkage group LG23.1 was assigned to LG 23.)

**FIGURE 4:**
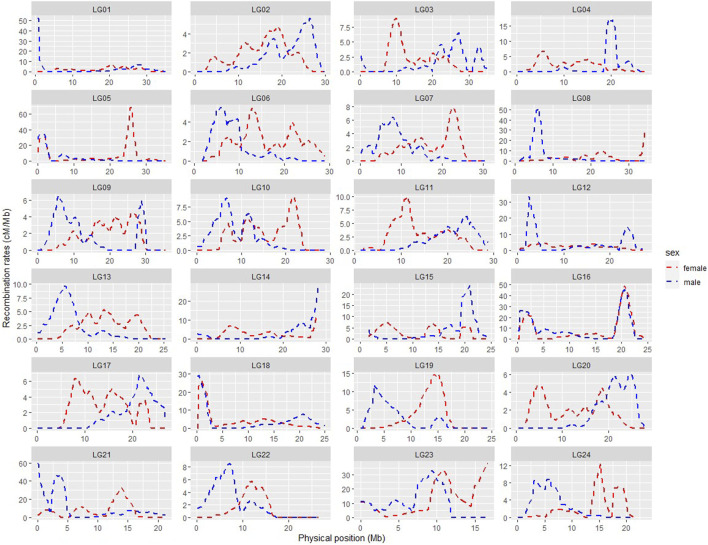
Comparison of recombination rate variation throughout the genome in the integrated female (red) and male (blue) maps.

SNP position information based on the *ASM435267v1* genome assembly was used to produce the physically informed consensus, female and male linkage maps (Supplementary Figure S2). A summary of the integrated maps is shown in Supplementary Table S3. A comparison of map positions between the integrated genetic and physical maps for different LGs is shown in Figure 3, in which most LGs exhibited sigmoidal patterns of recombination, with greater recombination rates toward the middle and low recombination rates toward the ends of the chromosomes. Large gaps or jumps can be seen in some of the plots, viewed from the *x*-axis or from the *y*-axis (Supplementary Table S4). Viewed from the *x*-axis, representing the physical position, large gaps were observed on LG24 (2.12 Mb) and LG1 (1.9 Mb), whereas viewed from the *y*-axis, representing the genetic position, large gaps were observed e.g., in LG4 (36.36 cM) of the integrated male map and in LG21 (50.37 cM) of the integrated female map. The markedly large jump downward in LG23 of the integrated consensus map was due to the fragmented linkage group LG23.1 assigned to LG23 in this case. Recombination rates of the three integrated maps are shown in Supplementary Table S3, and the recombination rate variation comparison of integrated female and male maps is visualised in Figure 4. The average recombination rate in the female was 3.55 cM/Mb whereas it in the male was 3.05 cM/Mb. The pattern of the recombination rates was different between male and female in some LGs, as there was a higher recombination rate for the male than for the female in the beginning of some LGs (e.g., LG09), whereas in other LGs the pattern was just opposite (e.g., LG20).

## Discussion

### Linkage Map Construction and Collinearity Analysis

The total genetic length of the consensus linkage map in our study was 1757.4 cM. The genetic map length (1885.67 cM) using the Mindong strain only, found by Kong et al. (2019), is slightly larger than that of our study. However, the linkage map length, also using Mindong strain, found by Ao et al. (2015) was 5451.3 cM, is much larger than in our study. The differences in total genetic length could be caused by the mapping family used in our study, which was a cross between Mindong strain and Daiqu strain. Suppressed recombination rates have also been reported in rainbow-Yellowstone cutthroat trout (*Oncorhynchus clarkii bouvieri*) hybrids, there explained by chromosome rearrangements (Ostberg et al., 2013). And in a hybrid cross of Human Pathogenic Fungus, *Cryptococcus neoformans*, the linkage map length (197 cM) was much shorter than those (1,356.3 cM) observed in a single strain (Sun and Xu, 2007).

The average female-to-male map length ratio was 1.2 ± 0.23 in our study, indicating more recombination events happening in females, which is consistent with an earlier study in large yellow croaker by Ning et al. (2007). The phenomenon of heterochiasmy, i.e., sex differences in recombination rates between the two sexes, has been found in many fish species. Higher recombination rate in female fish, as in our case, was also reported in Atlantic salmon (1.38) (Lien et al., 2011), gilthead sea bream (1.61) (Tsigenopoulos et al., 2014), Nile tilapia (1.2) (Joshi et al., 2018) and Gasterosteus sticklebacks (1.64) (Sardell et al., 2018), where in all cases it seems that the heterogametic sex has lower recombination rates. In our study, most LGs in the female map were larger than those in the male map, whereas the male map was larger in LG4, LG19, LG21 and LG24. Similar cases were also found in other fish, such as the gilthead seabream and Nile tilapia (Tsigenopoulos et al., 2014; Joshi et al., 2018). The molecular mechanisms for the sex differences in recombination rates are still not well understood. The differences may be caused by sexually antagonistic selection, meiotic drive in females, selection during the haploid phase of the life cycle, selection against aneuploidy, or mechanistic constraints; however, no single hypothesis can adequately explain the evolution of heterochiasmy in all cases (Sardell and Kirkpatrick, 2020).

Sigmodal patterns of recombination in large yellow croaker, with greater recombination rates toward the middle and lower recombination rates toward the ends, have also been seen in other species, like Nile tilapia (Joshi et al., 2018), whereas, salmon, channel catfish, etc (Li et al., 2014; Tsai et al., 2015) have shown opposite patterns, with higher recombination at the end of the LGs. The segments with little or no recombination may suggest possible location of centromeres. The karyotypes of large yellow croaker were earlier categorised into 10 pairs of sub-telocentric and 14 pairs of acrocentric chromosomes (Xu et al., 2017), implying that the centromeres are located at the end of the chromosomes, matching the low recombination rates seen towards the end of these LGs in our study. Recombination rate profiles within each LG also differed between males and female, with distinct regions containing potential recombination hotspots.

Physical gaps, as viewed from the *x*-axis in Figure 3, indicate lack of SNPs in these regions. One reason for these gaps could be massive repeat sites, unrecognisable by the *Pst I* enzyme during RADseq. Identifying additional markers with a different enzyme should thus help to fill these gaps. Another related reason could be the random and consequently partly uneven distribution of detected markers across the genomes, which is a disadvantage of RAD based technologies. Thus, RADseq usually generates medium density SNP linkage maps, leading to a low genome coverage (Robledo et al., 2017). Furthermore, the strain used in the *ASM435267v1* genome assembly, called DH2-L1, is a double haploid obtained by artificial gynogenesis from the Mindong strain only (Cai et al., 2010), whereas the population used in our genetic map is a cross between Daiqu strain and Mindong strain. The strain difference could thus be another reason for the physical gaps, as chromosomal rearrangements, including deletions, duplications, inversions, and translocations, could be different among strains.

Large jumps in some of the LGs were also viewed from the *y*-axis in Figure 3. These regions, with significantly elevated recombination rates, may be due to recombination hotspots, insufficient SNP coverage caused by the randomness of RAD sequencing explained above, and/or low level of polymorphism in the F_1_ family. A similar problem of large intervals was also presented in the genetic linkage map of the small yellow croaker (*Larimichthys polyactis*), also from only one fullsib family (Liu et al., 2020). Thus, use of multiple fullsib or halfsib families should be preferred, as done for instance with the high-density linkage map developed in Nile tilapia using 41 fullsib families (Joshi et al., 2018).

### QTL Analysis and GWAS for Growth Traits

QTL analysis and GWAS are two types of strategies to detect potential causal genes for quantitative traits. QTL analysis detect associations between marker intervals and phenotypes, while GWAS identifies associations between single DNA markers and phenotypes, and thus the two methods complement each other (Sonah et al., 2015).

For fish less than 10 months of age, the gonads are hard to assess only by naked eye observation and there is hardly any gender difference to use for sex determination (Wang and Cai, 2018). Thus, no gender information was available for the fish at 6 months in the present study. Growth differences have already been observed between the Mindong and Daiqu strains and some phenotype segregation may be expected in the F_1_, but no significant QTLs or SNPs were detected in our QTL or GWAS analysis. This was probably due to the complex genetic nature of the three growth traits which generally have been found to be controlled by many genes, each with minor effects. Also, the power of QTL analysis and GWAS will often not be sufficient with only one test family, due to the categorical nature of QTLs, for which a significant variant may or may not be present in any given family. For instance, the highly significant QTL variant that induced high resistance to IPN virus in the study of Moen et al. (2009), was only present in ca 5 % of the breeding nucleus. The QTL plot of BH is close to the threshold by 1,000 permutations, while the Manhattan plot of BH is far from the suggestive threshold by Bonferroni correction, which has been reported to be overly conservative in some cases (Kaler and Purcell, 2019). Also, no SNPs were identified to be significantly associated with the BL/BD ratio or the BL/BH ratio in large yellow croaker (Dong et al., 2019; Zhou et al., 2019). This may be due to low power in all these studies, but the results correspond well with the assumed polygenic nature of these traits. However, Xiao et al. (2015) identified several potential QTLs for growth traits (total weight, total length and total height) by composite interval mapping using 72 individuals from one fullsib family. But LOD score significance thresholds were not given in the plots in this study.

Using one F_1_ fullsib family, as in the present study, Kong et al. (2019) identified seven significant QTLs linked to white spot disease resistance. The probability of identifying the QTLs in disease resistance traits could be higher than in growth traits, as it is often found that they are controlled by some major QTLs (Fraslin et al., 2020). However, these studies, using one F_1_ family, only provide preliminary results of QTL mapping, and studies involving a more representative sample of the breeding population are required to conduct a marker-assisted selection scheme. One fullsib family is thus not ideal for identifying candidate genes, and a larger sample size and more families should be used to improve the power and to reveal potential associations (Korte and Farlow, 2013).

## Conclusion

A consensus genetic linkage map for large yellow croaker was constructed with 20,147 SNPs from RAD sequencing, based on an F_1_ family from Mindong strain and Daiqu strain. The total length of the consensus map was 1757.4 cM with an average marker interval of 0.09 cM. The female-to-male linkage map length ratio was 1.2. The map was adjusted based on the physical map, and integrated consensus and sex-specific linkage maps were generated. The recombination pattern mostly showed sigmoidal pattern of recombination. In most LGs, higher recombination rates were found in the integrated female map, compared to the integrated male map. No significant QTLs for growth related traits in fish at 6 months were found, probably due to the low detection power in only one family and the polygenic and complex nature of growth traits that are controlled by many genes with minor efforts. The present study indicates that there may be genetic differences between the two strains Daiqu and Mindong, which may have implications for breeding programs using DNA-information in a future selection scheme.

## Data Availability

The sequenced data used for SNP detection has been deposited in Sequence Read Archive in NCBI (PRJNA786283). The datasets presented in this study can be found in online repositories. The names of the repository/repositories and accession number(s) can be found below: https://figshare.com/, doi: 10.6084/m9. figshare.16779160.
